# Effect of Essential Oils Supplemented with Caprylic Acid and Sodium Chloride against Faecal ESBL-Producing *Escherichia coli* Isolated from Pigs

**DOI:** 10.3390/antibiotics11040461

**Published:** 2022-03-29

**Authors:** Daiga Gāliņa, Vitalijs Radenkovs, Jorens Kviesis, Anda Valdovska

**Affiliations:** 1 Faculty of Veterinary Medicine, Latvia University of Life Sciences and Technologies, K. Helmaņa iela 8, LV-3004 Jelgava, Latvia; anda.valdovska@llu.lv; 2 Research Laboratory of Biotechnology, Latvia University of Life Sciences and Technologies, Lielā iela 2, LV-3001 Jelgava, Latvia; vitalijs.radenkovs@llu.lv; 3 Department of Environmental Science, Faculty of Geography and Earth Sciences, University of Latvia, Jelgavas iela 1, LV-1004 Rīga, Latvia; jorens.kviesis@lu.lv

**Keywords:** *Thymus vulgaris*, *Thymus serpyllum*, *Satureja montana*, MDR, antibacterial activity, MIC, MBC

## Abstract

The purpose of the present investigation was to compare the antibacterial activity of six commercial and lab-scale extracted essential oils (EOs) alone or in combination with caprylic acid (CA) and sodium chloride (NaCl) against faecal *Escherichia coli* with and without extended-spectrum beta-lactamase (ESBL) encoding genes, and of isolates classified as multidrug-resistant (MDR). Gas chromatography–mass spectrometry (GC–MS) was used for the analysis of chemical composition of EOs, while the minimum inhibitory concentration (MIC) and minimum bactericidal concentration (MBC) assays were carried out to elucidate the antibacterial activity of non-supplemented and supplemented EOs against different resistance levels of *E. coli* strains. The main compounds in commercial EOs were aromatic monoterpenoids (30–56%) and *p*-cymene (8–35%), while the main compounds in the lab-scale EOs were aromatic monoterpenoids (12–37%) and *γ*-terpinene (18–22%). Commercial EOs exhibited superior inhibitory activity of *E. coli* in comparison to lab-scale produced EOs. Antibacterial activity of EOs was significantly enhanced by enrichment of the EOs with NaCl (*p* < 0.001) or CA (*p* = 0.012). Most of the non-supplemented EOs exhibited lower activity against MDR and ESBL producing *E. coli.* In contrast, EOs supplemented with CA and especially NaCl was equally effective against ESBL and non-ESBL as well as MDR and non-MDR *E. coli.* It was found that supplementation of EOs with NaCl could enhance the antibacterial activity towards ESBL and MDR *E. coli* isolates. However, additional studies are needed to clarify the potential risks of developing resistance.

## 1. Introduction

Among many global threats in this century, the relevance for preventing and reducing the spread of antimicrobial resistance continues. The misuse and overuse of antibiotics in food-production animals are resulting in a selection of resistant bacteria. Particular attention has been paid to extended-spectrum beta-lactamase (ESBL) producing bacteria, which coffer resistance to a range of critically important antibiotics in human medicine and since beta-lactamase (BLA) genes are commonly located in the mobile genetic elements together with other resistance genes, they often provide co-resistance to other antibiotics [1]. According to the report, *Global Trends*, pig farming is one of the highest consumptions of antibiotics by animals [2]. There has been growing emphasis on reducing the use of antibiotics and synthetic antimicrobials in pig farming by finding effective, safe, and natural origin alternatives such as feed additives or disinfectants that particularly could compete with already existing synthetic ones.

Essential oils (EOs) are considered to have great potential due to their natural origin, low toxicity, and minimal adverse effects [3]. In addition, numerous studies have proven the antimicrobial activity of EOs on various pathogens [4]. The relatively high antibacterial effect has been achieved with the EOs derived from the Lamiaceae family. The antibacterial activity of EOs attributed primarily to the presence of such monoterpenoid phenols as thymol or carvacrol [5]. Both of them are derivatives of cymene, structural isomers with different locations of the hydroxyl groups on the phenolic ring. Their hydrophobic properties and availability of free hydroxyl groups explain a superior antimicrobial activity [6,7]. The hydrophobic compounds in EOs facilitate affinity and accumulation between the fatty acid chains of the lipid bilayer into the bacterial cell membrane and decrease membrane integrity [7]. Delocalised electron systems and hydroxyl groups are capable of reducing pH and the potential of the membrane by activating the proton exchange, consequently reducing ATP synthesis and resulting in cell death [7,8]. It has been reported that structural conformation and the number of functional groups in the molecule, such as OH, define the antimicrobial activity of a particular compound [9].

In recent years, fatty acids have been identified as the next generation of antimicrobials, especially due to their unconventional mechanisms for action, such as inhibition of horizontal gene transfer, quorum sensing, and efflux pumps of bacteria, thus opening up opportunities to reduce the development of bacterial resistance and virulence [10]. The safety and broad spectrum of activities attributed to medium-chain fatty acids (MCFAs) have tempted to use it as antibiotic alternatives, especially in the food and animal industry where the use of antibiotics is prohibited or banned [11]. Caprylic acid (C8:0) is one of the MCFA and belongs to the family of saturated fatty acids. More recent studies have shown that this representative of MCFA has been used as a component of some intravenously administered total parenteral nutrition formulations [12,13]. Synergistic antimicrobial activity of CA with citric acid has been observed by [14], demonstrating positive inhibition of *E. coli* O157:H7. The ability of CA to pose as an effective antimicrobial agent is due to its structure, molecular size, and pK. Under a protonated state, CA begins interacting with the lipophilic fraction presented in microbial cell membranes, promptly penetrating through the cell membranes after and causing disruption of its integrity [15]. However, to achieve sufficient antimicrobial effect, a relatively high concentration of CA is required [16].

Sodium chloride (NaCl) is widely used in the food industry as an additive to flavour and preserve food products. A high concentration of NaCl reduces water activity and increases osmotic pressure in medium, therefore causing stresses in microorganisms and suppressing their growth [17]. On the one hand, there are concerns about the osmotic stress-induced salt overly sensitive (SOS) response of bacteria that could be the main mechanism for horizontal gene transfer [18]. Meanwhile, high salinity promotes the elimination of resistant plasmids, thus reducing the relative amount of antibiotic-resistant genes in the medium of high salt concentration [19]. The synergistic effect of using EOs together with additives of natural origin is considered a safer and more effective approach to address the concerns for the spread of resistant bacteria [20]. Moreover, the synergistic effect among EOs and additives will reduce the effective enquired dose required to cause damage to resistant bacteria.

Although EOs have been extensively studied, there is still a limitation in comparative information on the antimicrobial activity of EOs extracted from the same plant species grown in the southern and northern regions of Europe (Latvia). Meanwhile, it is essential to provide additional information on the antimicrobial activity of EOs coupled with well-known antimicrobial agents to suppress the proliferation of bacterial isolates with different levels of resistance, depending on the genes acquired or non-acquired. This not only could facilitate the practical use of EOs supplemented with additives of natural origin, but also recognise potential threats of resistance. Therefore, the purpose of the present investigation was to compare the antibacterial activity of six EOs recovered from Lamiaceae family plants grown in northern Europe (Latvia) and commercially available EOs of southern Europe (Spain, Croatia, and Turkey) origin against faecal *E. coli*, isolated from Duroc–Landrace cross-breed pigs (*Sus scrofa domesticus*), and to evaluate the antibacterial activity of EOs supplemented with CA and NaCl against faecal *E. coli* with and without ESBL encoding genes and the isolates classified as MDR.

## 2. Results

### 2.1. Chemical Composition of Essential Oils

A relatively large variation in the profile and concentrations of volatiles was observed between the commercial and lab-scale prepared EOs of selected plant material. In total, 69 volatile compounds were identified in the EO of *T. serpyllum* originating from northern Europe (NEU), while 38 were identified in the industrially produced EO of *T. vulgaris* from the southern part of Europe (SEU). Generally, commercial EOs had a higher percentage of aromatic monoterpenoids, e.g., thymol and carvacrol, which accounted for 30–56% of the total aroma volatiles. In turn, EOs obtained under lab-scale conditions from plant species grown under NEU conditions contained a considerably lower amount of thymol and carvacrol, corresponding to 12–37%. The percentage of the total monoterpenoids in *T. vulgaris* EOs was found to be similar between SEU and NEU regions, corresponding to 30 and 31%, respectively. A higher level of monoterpene hydrocarbons was observed in both *T. serpyllum* (2446 mg/kg) and *S. montana* (2302 mg/kg) EOs from NEU. Moreover, all EOs of this group demonstrated a considerably higher abundance of *γ*-terpinene (17.83–22.42%) compared to commercial EOs (5.81–9.24%) (Table 1).

The results revealed that the main representatives of the commercial *T. vulgaris* EO were *p*-cymene (35.4%), thymol (26.66%), *β*-linalool (7.31%), and *γ*-terpinene (5.81%). In contrast, the main components detected in *T. vulgaris* EO originated from NEU were thymol (27.59%), *p*-cymene, (23.63%), and *γ*-terpinene (17.83%). The main components of commercial *S. montana* EO were found to be carvacrol (26.37%), thymol (17.10%), *β*-caryophyllene (14.37%), *p*-cymene (13.02%), and *γ*-terpinene (6.64%), while they were carvacrol (35.54%), *γ*-terpinene (22.42%), and *p*-cymene (10.58%) for the lab-scale produced EO. A considerable difference in both profile and concentrations of volatiles was observed between commercial and lab-scale produced EOs of *T. serpyllum*. As seen, the main representatives of SEU were carvacrol (53.34%), *γ*-terpinene (9.24%), and *p*-cymene (8.18%), while they were *γ*-terpinene (21.93%), *β*-caryophyllene (12.49%), *p*-cymene (8.94%), *trans*-geraniol (8.37%), and carvacrol (7.70%) for NEU.

### 2.2. Antibacterial Activity of Commercial and Latvian EOs

According to the antibacterial activity results of all six EOs, no significant differences were observed between the values of minimum inhibitory concentration (MIC) and minimum bactericidal concentration (MBC at 95% and 99.5%) (Table 2).

**Figure 1:**
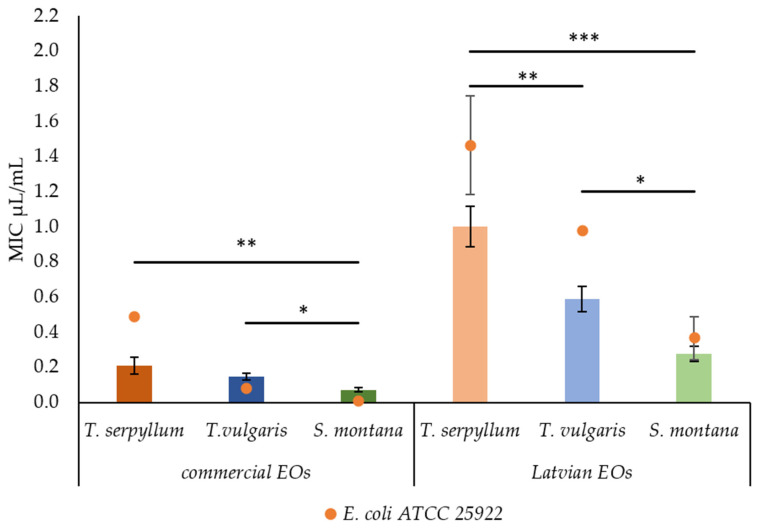
Comparison of minimum inhibitory concentration (MIC) by Latvian and commercial essential oils against reference *E. coli* ATCC 25922 and faecal *E. coli*. * *p* < 0.05; ** *p* < 0.01; *** *p* < 0.001.

The findings revealed, however, that the origin and the plant species have a significant effect on the antibacterial activity of EOs against faecal *E. coli* isolated from pigs. NEU lab-scale produced EOs had a higher (*p* < 0.001) value of MIC compared to the commercial ones. Moreover, significant differences were observed among the plant species. The most effective EO was found to be *S. montana* EO, while the least was found to be *T. serpyllum* EO. The differences (*p* = 0.002) in antibacterial activity among commercial EOs were also revealed. The most effective antibacterial activity was observed for *S. montana* EO, while no significant differences were found between the EOs of *T. serpyllum* EO and *T vulgaris* (Figure 1).

The highest antibacterial activity was observed for EOs recovered from *S. montana*; however, commercial EO demonstrated significantly higher (*p* = 0.013) activity than that of lab-scale origin. Interestingly, the values of MIC and MBC of NEU-originated EOs were higher than the reference *E. coli* strain compared to faecal *E. coli*, isolated from pigs. For commercial EOs, such ability was observed only in *T. serpyllum* EO.

### 2.3. Effect of Supplementation of Commercial EOs with Caprylic Acid and Sodium Chloride on Antibacterial Activity against Faecal E. coli

Initial screening of antibacterial activity of supplements used in this research, i.e., 1 mM CA or 3% NaCl revealed no considerable inhibitory activity against any of *E. coli* isolates selected. At the same time, supplementation of commercial EOs with CA and 3% NaCl resulted in significant enhancement of the antibacterial activity against faecal *E. coli*. Antibacterial activity was increased substantially (*p* < 0.001) by enrichment of EOs with 3% NaCl. The positive effect (*p* = 0.012) was observed for EOs supplemented with CA. Comparing the antibacterial activity of EO plus CA and EO plus 3% NaCl, no significant differences were observed, while only a tendency of salt (*p* = 0.078) to be more effective against faecal *E. coli* than CA was highlighted.

**Figure 2:**
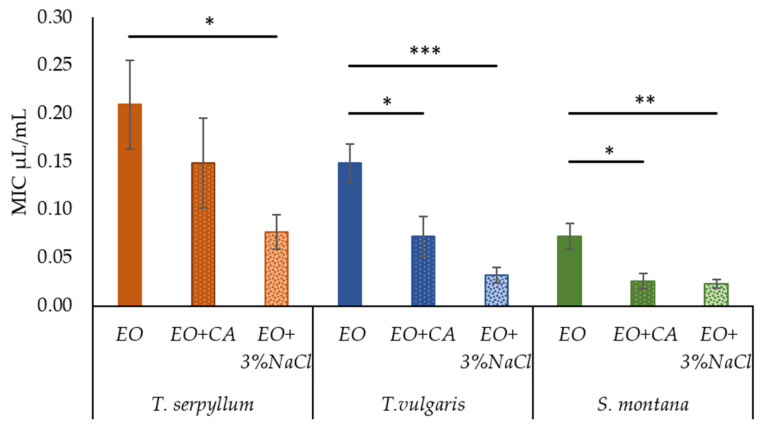
The effect of caprylic acid and 3% NaCl on the antibacterial activity of commercial essential oils (*T. sepryllum*, *T. vulgaris* and *S. montana*) against faecal *E. coli*. * *p* < 0.05; ** *p* < 0.01; *** *p* < 0.001.

In the supplementation of *T. serpyllum* EO with CA, the value of MIC reduced by 29%, while in the case of EO enrichment with 3% NaCl, the MIC value reduced by 63% (from 0.209 ± 0.046 to 0.077 ± 0.018 μL/mL). Supplementation of *T. vulgaris* EO with CA or 3% NaCl, significantly reduced the value of MIC by 51% (from 0.148 ± 0.020 to 0.072 ± 0.020 μL/mL) and 78% (from 0.148 ± 0.020 to 0.032 ± 0.009 μL/mL), respectively. Supplementation of EOs derived from *S. montana* with CA or 3% NaCl resulted in a significant increase in antibacterial activity by ensuring the reduction in MIC values by 64% (0.073 ± 0.014 to 0.023 ± 0.008 μL/mL) and 69% (0.073 ± 0.014 to 0.023 ± 0.005 μL/mL), respectively (Figure 2).

### 2.4. Comparison of Antibacterial Activity of Essential Oils with and without Supplementation against Faecal ESBL-Producing and Non-ESBL-Producing E. coli

In general, 3% NaCl as a supplement to EO significantly promoted (*p* < 0.001) the decrease in MIC value against ESBL-producing *E. coli*, while CA only had a tendency (*p* = 0.10) to decrease. Based on the results, the obvious superiority of EOs recovered from the plant species of *S. montana* and *T. vulgaris* over *T. serpyllum* was confirmed statistically (*p* < 0.001).

**Figure 3:**
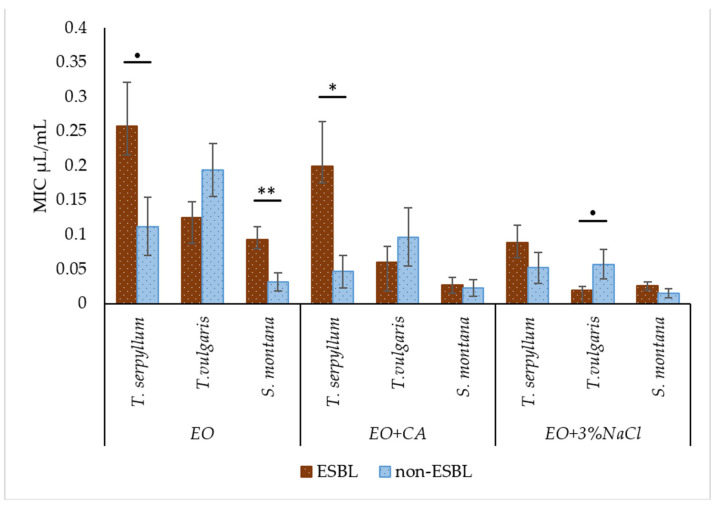
Comparison of antibacterial activity of *T. serpyllum*, *T. vulgaris* and *S. montana* against ESBL-producing *E. coli* and non-ESBL-producing *E. coli*, depending on supplemented groups: essential oils (EO), essential oils supplemented with caprylic acid (EO + CA) and essential oils supplemented with sodium chloride (EO + 3% NaCl). ^●^ *p* = 0.05–0.10; * *p* < 0.05; ** *p* < 0.01.

Some of the non-supplemented EOs and those enriched with additives demonstrated significant differences in antibacterial activity or different trends were highlighted against ESBL and non-ESBL-producing *E. coli*. It was revealed that non-enriched EOs of *T. serpyllum* and *S. montana*, as well *T. serpyllum* EO supplemented with CA had the highest MIC values against ESBL-producing *E. coli*. In contrast, EOs derived from *T. vulgaris* from the group EO plus 3% NaCl had a tendency to show a lower value of MIC against ESBL-producing *E. coli*. However, in most cases enriched EO was equally effective against ESBL and non-ESBL producing *E. coli* (Figure 3).

### 2.5. Comparison of Antibacterial Activity of Essential Oils with and without Supplementation against Faecal MDR and Non-MDR E. coli

The supplementation of EOs with CA and 3% NaCl significantly decreased (*p* < 0.001) the MIC values against MDR *E. coli* compared to non-supplemented EOs. The factor ‘plant species’ had no significant effect on increasing the activity against MDR *E. coli* in each supplemented group, except for the EO plus 3% NaCl group, the *T. vulgaris* had a tendency to be more effective (*p* = 0.013) than *T. serpyllum*.

**Figure 4:**
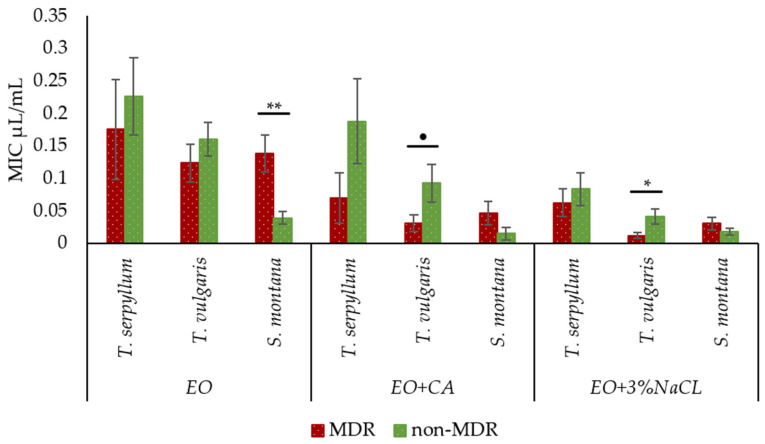
Comparison of antibacterial activity of *T. serpyllum*, *T. vulgaris* and *S. montana* against MDR *E. coli* and non-MDR *E. coli*, depending on supplemented groups: essential oils (EO), essential oils supplemented with caprylic acid (CA + CA), and essential oils supplemented with sodium chloride (EO + 3% NaCl). ^●^ *p* = 0.05–0.10; * *p* < 0.05; ** *p* < 0.01.

Comparing the antibacterial activity of each plant species (with and without CA and 3% NaCl supplementation) against faecal MDR and no-MDR *E. coli*, non-supplemented *S. montana* EO demonstrated a significantly higher MIC value against MDR *E. coli.* Interestingly, in the supplemented groups, *T. vulgaris* EO was more effective against MDR *E. coli* than non-MDR *E. coli.* The MIC value of *T. vulgaris* EO plus CA against MDR and non-MDR *E. coli* were 0.03 ± 0.013 and 0.09 ± 0.029 μL/mL, respectively, and 0.01 ± 0.005 and 0.04 ± 0.012 μL/mL in case of *T. vulgaris* EO plus 3% NaCl, respectively. The other EOs supplemented with CA and NaCl were equally effective against MDR and non-MDR (Figure 4).

## 3. Discussion

Plants from the Lamiaceae family due to abundance in aroma volatiles have been used in traditional medicine for centuries in treating various ailments, including gastrointestinal disorders, infections, dermatitis, bronchitis, and inflammation [21]. *T. serpyllum* EOs of SEU are often classified as thymol chemotype, due to the high concentration of thymol [22,23]. The dominance of thymol in EOs of *S. serpyllum* plants grown under southern latitude has been highlighted by a research group from Serbia, indicating the percentage distribution of volatiles as follows: thymol (38.5%), *p*-cymene (8.9%), and *γ*-terpinene (7.2%) [23]. A similar observation has been made by another research group, highlighting thymol (54.17%), *γ*-terpinene (22.18%), and *p*-cymene (16.66%) as the main representatives of EOs recovered from *S. serpyllum* [22]. However, based on the results of the present study, the main representative of aroma volatiles detected in commercial EOs of *T. serpyllum* was found to be carvacrol (53.34%) and only 1% thymol.

Some authors have reported the presence of mixed chemotypes of *T. serpyllum*, e.g., in the EO from Slovakia, the concentration of thymol and carvacrol corresponded to 18.8% and 17.4%, respectively [24]. In the EO of *T. serpyllum*, the carvacrol chemotype was reported quite rarely in another study [25]. The percentages of thymol and carvacrol observed in Latvian *T. serpyllum* EO were 3.74% and 7.70%, which is lower than observed in commercial products, respectively. Though, the amounts were notably higher than reported by the Lithuanian colleagues, where the concentrations of thymol and carvacrol in the EOs did not exceed 2.3% and 2.0, respectively [26]. This observation was reinforced by the research group from Estonia, indicating that the values of thymol and carvacrol recovered from *S. serpyllum* did not exceed 2.9% and 3.5%, respectively [27]. However, the results of the current research are consistent with those obtained by Paaver et al. [28], indicating similar carvacrol and thymol percentages in the commercial *T. serpyllum* EOs purchased from retail pharmacies in Latvia. The content of carvacrol and thymol was found to be 11.5% and 3.0%, respectively.

As seen, slight differences in the main components were found between NEU and SEU EOs from the *T. vulgaris* plant species. The content of thymol was 27.59% (NEU) and 26.66% (SEU). The most significant difference in the thymol content was observed between the EOs derived from *S. montana*, with commercial SEU EOs having the highest content (17.10%) and the lowest (1.41%) for the lab-scale NEU. The lowest content of thymol was found in EOs recovered from *T. serpyllum* in NEU (3.74%) and even lower in the commercial SEU sample (1.00%).

The *p*-cymene was the second highest representative of aroma volatiles, the relative availability of which was confirmed by the GC–MS. The highest content of this compound was found in EOs recovered from *T. vulgaris*, with significant differences between the commercial SEU (35.4%) and lab-scale NEU (23.63%) groups. Considerably lower values of *p*-cymene were detected in *S. montana*, with commercial SEU EOs having the highest content (13.02%) and lab-scale NEU the lowest (10.58%). Similar to thymol, the content of *p*-cymene in *T. serpyllum* EOs both in SEU and NEU was found to be the lowest, however, with no significant difference between the groups.

However, it should be noted that *γ*-terpinene and *β*-linalool are the main compounds in lab-scale (NEU) and commercial (SEU) EOs, respectively, the dominance of which was also confirmed statistically. Thymol chemotype was the most widely reported chemotype for EO of *T. vulgaris*, while *p*-cymene, *γ*-terpinene, and *β*-linalool were equally often observed in variable precentral quantities as representatives of aroma volatiles in *T. vulgaris* EOs [22,23,29,30].

Carvacrol was identified as another compound abundantly present both in commercial (SEU) and lab-scale (NEU) *S. montana* and *T. serpyllum* EOs. This observation is in line with data reported earlier, though, demonstrating at the same time relative percentage fluctuations from 13.7% to 76.6% depending on the origin of the plant [31,32,33]. Less commonly reported chemotypes for *S*. *montana* EOs were *p*-cymene-linalool [34], and *β*-caryophyllene-germacrene D [35]. In general, lower concentrations of thymol and carvacrol were observed in lab-scale NEU extracted EOs. Although, Ložienė et al. reported that the northern climate could negatively affect the development of volatile phenolic compounds in considerable quantities, thus explaining the lower amount of individual compounds observed in the NEU group [26]. At the same time, almost an equivalent amount of total aroma volatiles was estimated for NEU and SEU EOs recovered from *T. vulgaris*. Moreover, a remarkable amount of carvacrol was observed in *T. serpyllum* grown under NEU. A relatively high concentration of thymol (75.8–80.5%) was observed in EOs derived from *T. vulgaris* grown in north Finland [30]. In addition, large variations of volatile aroma compounds in *S. montana* EOs were reported in one SEU country [35]. This suggests that genetics of plants rather than climate could influence the synthesis of aroma volatiles in plants thereby affecting the composition and content of aroma volatiles in plants.

The present study revealed the abundance of non-oxygenated hydrocarbons in the lab-scale extracted EOs compared to that contained in the commercial samples, which presumably resulted in a reduction in antibacterial activity. It has been indicated that the presence of *γ*-terpinene, in particular, could serve as an indicator for weak antibacterial activity [36]. This phenomenon could be explained by the ability of non-oxygenated monoterpenes to reduce the aqueous terpene solubility and exhibit an antagonistic effect on the oxygenated monoterpene hydrocarbons [37]. In turn, the presence of monoterpene *p*-cymene in high concentrations in commercial EOs contributed to a greater extent to antibacterial activity. The synergic effect of the *p*-cymene and carvacrol has been confirmed by Ultee et al. [8]. The *p*-cymene and its hydrophobic properties promote penetration of carvacrol through bacterial cell membranes thereby ensuring the inhibitory activity of carvacrol and destabilising cells membranes [8,38]. This feature of *p*-cymene has been confirmed by [39], indicating the potential contribution of this molecule as an enhancer to transdermal formulations for facilitating the penetration of drugs into human skin.

Regardless of whether Latvian and commercial EOs from *T. vulgaris* demonstrated almost equivalent amounts of aromatic, oxygenated, and non-oxygenated monoterpenes with non-significant differences in individual components, there were considerable differences in antibacterial activity of the EOs tested. This finding reveals the importance of small molecules as possible contributors to antibacterial activity. Commercial EOs recovered from *T. vulgaris* contained a remarkable amount of limonene and *β*-linalool. Limonene is one of the few non-oxygenated monoterpenes that has demonstrated considerable antibacterial activity (0.25 mg/mL) against several bacteria, incl., *E. coli* [36]. Moreover, *β*-linalool has well-established antibacterial activity against *E. coli* and Gram positive bacteria [36,40]. Presumably, the superior antibacterial activity of commercial EOs derived from *T. vulgaris* could be associated with the synergy of thymol and *β*-linalool and/or limonene as was mentioned in the case of *p*-cymene and carvacrol.

Surprisingly, in spite of the relative abundance of carvacrol in the commercial EO of *T. serpyllum* (2334 mg/kg), the antibacterial activity was lower than expected. This phenomenon was also observed by [25], proposing an antagonistic effect of some individual compounds presented in the EOs. Gallucci et al. observed an antagonistic effect of carvacrol and *β*-myrcene against *E. coli* and Gram positive bacteria [41]. It should be emphasised that the highest amount of *β*-myrcene was found in the commercial *T. serpyllum* EO.

The findings of the present study are in agreement with previous research indicating the positive interaction of EOs or phenolic compounds in EOs and medium-chain fatty acids (MCFAs) with enhanced antimicrobial activity of EOs [16,42,43]. The antibacterial activity of fatty acids is explained by their interaction with the bacterial cell membranes [44]. Amphipathic properties of fatty acids promote the formation of so-called lipid micelle aggregates through interactions of the lipophilic tail of fatty acids with phospholipids of the bacterial membrane that results in disruption of cell membranes’ integrity and causing its further oxidation [10,11]. In addition, fatty acids have the ability to disrupt electron transfer reactions by binding to the electron carriers, thereby preventing cell energy production while decreasing the membrane potential by interfering with oxidative phosphorylation [11,44]. It is worth noting that the MCFAs are considered to be much more active against Gram positive bacteria even at low concentrations; however, relatively high concentrations of these compounds are required for inhibition or to cause bacterial membrane lysis of Gram negative bacteria [16]. The synergistic activity of MCFAs and EOs has been studied before [16,42,43], demonstrating MCFAs inhibition activity enhancement at lower concentrations through the addition of various plant-derived EOs. High synergistic bactericidal activity has been observed by using MCFAs together with either carvacrol or thymol [16,43]. Furthermore, the most evident alteration in membrane potential has been recorded, especially for Gram negative bacteria applying a mixture of carvacrol and CA [16]. The reduced potential of the membrane destabilises the integrity, which causes an increase in its permeability [16]. The damages induced by MCFAs promote the penetration of hydrogen ions, which have a strong bactericidal effect [43]. The synergistic effect of CA in combination with EOs allowed for the successful inhibition of *E. coli* by natural compounds from plant origin. Supplemented *T*. *serpyllum* EO with CA was the only combination where no significant effect of supplementation was observed. However, supplementing the EO with CA allowed for a reduction in the amount of EO by 29%, at the same time not losing antibacterial activity. Fewer antibacterial activity of this combination could be associated with interactions between all of the aroma volatiles present in this EO.

Considering the results of the present study, *E. coli* may successfully survive and grow under an environment of elevated osmolality (3% NaCl). However, through the application of a mixture of 3% NaCl with EOs, increased antibacterial effect against faecal *E. coli* could be achieved. Kim et al., has studied the susceptibility of bacteria to different NaCl concentrations in the range 3–15%, also evaluating the degree of cell membrane damages. It has been found that the degree of damages negatively correlated with the amount of NaCl used; however, damages were reversible when bacteria were cultivated on a nutrient medium [45]. A study on the effect of 5–10% NaCl on *E. coli* O157:H7 revealed that *E. coli* was able to maintain the integrity of its structure and still proliferate after 12 h of incubation; however, by prolonged exposure to salt stress for 24 h, some morphological changes became apparent, revealing that the ability of cells to cope with stress was exhausted [46]. The hypertonic environment of high external osmotic pressure induces water efflux and dehydration of bacteria [47]. The first rapid response to hyper-osmotic shock is the accumulation of inorganic ions (often K^+^) [48]. After exceeding the breakpoint of primary response, which is approximately V 0.5 M NaCl (0.52 M NaCl was used in the present study), a secondary response is triggered, i.e., active synthesis of osmoprotectants via osmoregulatory systems of bacteria [47,48]. Osmotic shock-induced water fluxes are a great challenge for bacteria [47] and therefore, the bacterial response to osmotic shock by diversion of bacterial energy to osmoregulatory systems may have contributed to the exposure of EOs. The hydrophobicity of aromatic monoterpenoids contributes to the accumulation in the phospholipid bilayer and results in increased permeability of the membrane [7]. In addition, the hydroxyl groups affect ion-exchange reaction and reduce vital ATF synthesis, resulting in stock of ATP reserve and inevitable cell death [7,8]. In the present study, the constant concentration of NaCl was applied; therefore, it was not possible to calculate the Fractional inhibitory concentration index (FICI) for confirmation of synergism between EOs and 3% NaCl. However, the results of the present study are consistent with other authors [45], indicating that the addition of NaCl significantly reduces the concentrations of EOs needed to reach sufficient antibacterial activity. In addition, in the above study, both combinations of carvacrol plus NaCl and thymol plus NaCl at the respective concentrations were equally effective in the inhibition of *E. coli* O157:H7.

A greater synergistic potential was observed for EOs that contained a high level of thymol; however, more in-depth studies with a larger number of isolates are needed to confirm this observation. Besides, not exclusively thymol, but also other components detected in lab-scale produced EOs, possessing antibacterial agents against the selected test bacteria. It is speculated that some of the aroma volatile compounds exhibited presumably act as antagonists making relatively active compounds less effective under mixture models.

There have been many reports confirming the effectiveness of EOs against ESBL-producing and/or MDR *E. coli* pathogens [16,22,49]. However, limited information is available so far, showing a comparison of the sensitivity of different levels of resistance, e.g., ESBL/non-ESBL and MDR/non-MDR *E. coli* strains with the same EOs. The results of the present study reveal that non-supplemented EOs (except *T. vulgaris* EO) have a higher MIC value against ESBL- compared to non-ESBL-producing *E. coli* (antibiotic sensitive strain). In turn, by comparing the efficacy of non-supplemented EOs against MDR and non-MDR *E*. *coli S. montana* EO was found to have a significantly higher MIC value against MDR *E. coli*. Benameur et al., reported that different activity of *T. vulgaris* EO against MDR ESBL-producing *E. coli* depends on containing *bla* genes–CTX-M-1 producing *E. coli* being more sensitive than SHV producing *E. coli* [49]. However, in our opinion, this should be approached with caution, as both strains are classified as MDR, so the potential impact of other genes should be considered. The resistance of ESBL-producing *E. coli* has not been only associated with the presence of hydrolysing beta-lactamases, but also with the reduced permeability of the outer membrane due to loss or modification of porins [50,51]. In addition, efflux pumps are a common mechanism for resistance in MDR bacteria. Furthermore, they have physiological functions, particularly in stress conditions, as a response to environmental and physiological signals [52]. In our opinion, decreased membrane permeability of ESBL-producing *E. coli* and the efflux pump presence of MDR *E. coli* could be the main reasons causing the lower activity of some pure EOs.

The ability of bacteria to develop resistance to EOs remains inconsistent. Some of the serovars of *Salmonella enterica* were reported to have the ability to adapt to the basil environment with the further development of resistance to the basil oil and its main compounds, i.e., linalool, estragole, and eugenol as reported for serovar of *S*. Senftenberg [53]. The following resistance mechanisms, such as selective permeability, reduced influx, and chemotaxis-controlled motility, were identified for linalool resistant *S. enterica* serovar Senftenberg mutants [54]. Prolonged exposure to linalool has exceedingly increased MIC value to this compound and facilitated cross-resistance against several antibiotics, yet at the same time these mutants may experience a significant challenge to survive in the environment [55]. However, it should be emphasised that the above findings on the role of EOs for the development of resistant bacteria are based on the single component of EO. Several authors acknowledge that bacteria rarely develop resistance to EOs due to the wide diversity in active compounds and several targets in the bacteria [56,57]. Along with the ability of EO to reduce expression of genes related to virulence factors of *S. aureus* and impair antioxidant system making them more susceptible to oxidant attack, the compounds present in EO could also affect the activation of SOS responses [58]. In turn, Al-Kandari et al. reported that thymol may induce the resistance of *E. coli* by genetic, morphological, and metabolic changes, but at the same time, it becomes uncompetitive in the environments, thus they may not raise the threat of resistance [59].

Interestingly, the supplementation of EOs either with CA or NaCl lowered differences in the sensitivity between ESBL and non-ESBL-producing *E. coli* as well as between MDR and non-MDR *E. coli*. Moreover, enriched *T. vulgaris* EO with NaCl was found to be more effective against resistant *E. coli* than sensitive strains. This suggests that multi-target exposure by enriched EOs may be more successful at bypassing the resistance mechanisms and survival of bacteria. Furthermore, a salty environment ensured by the incorporation of NaCl to EOs was found to be effective, since it causes morphological changes in the bacteria membranes and makes the surface more amorphous and readily permeable for bioactives to pass through. It has been reported that NaCl may reduce biofilm formation motile ability [60], while abiotic stress could promote the elimination and inhibition for the horizontal transfer of plasmids [19].

## 4. Materials and Methods

### 4.1. Essential Oils (EOs)

A total of six EOs were included in this study. Three of them were prepared under lab-scale conditions from plant materials grown under northern latitudes (Latvia) (NEU), while the other three were of commercial origin (SEU).

#### 4.1.1. Authors-Obtained Essential Oils

Plants used in this study, i.e., breckland thyme (*Thymus serpyllum* L.), common thyme (*Thymus vulgaris* L.), and mountain savory (*Satureja montana* L.) were collected in July 2019 at the full flowering stage. Authentication of plants was assured by the Institute of Horticulture. The *T. serpyllum* was collected in the area of Jelgava (56°39′45.4″ N 23°45′13.9″ E) and Salaspils (56°52′11.4″ N 24°21′04.5″ E), while *T. vulgaris* and *S. montana* were picked from a certified organic farm in the area of Kraslava (55°53′50.5″ N 27°05′55.6″ E).

Collected plants were subjected to hydrodistillation to obtain EOs using a Dean–Stark distillation apparatus as described by [61] with slight modifications. Briefly, freshly collected aerial parts of each plant were manually sliced into 1–1.5 cm pieces and 300–400 g of the plants together with 2 L of water were subjected to hydrodistillation for 1.5–2.5 h to extract EOs. Each hydrodistillation procedure was carried out until no more visible drops of oil were extracted. The yield of EOs recovered from *T. serpyllum*, *T. vulgaris* and *S. montana* were 0.67%, 0.73%, and 1.09% (*v*/*w*), respectively. The EOs were stored at −18 °C, in a dark place.

#### 4.1.2. Commercial Essential Oils

EO of *T. serpyllum* was purchased from the manufacturer Primavera (Oy-Mittelberg, Germany), the origin country–Turkey. *T. vulgaris* EO was acquired from the manufacturer Oils4life (Great Yarmouth, Norfolk, United Kingdom); the origin country was Spain. *S. montana* EO was obtained from the supplier Hermitage Oils (Areco, Tuscany, Italy); the origin country was Croatia.

#### 4.1.3. Preparation of Essential Oils for Analysis of Aroma Volatiles by Gas Chromatography–Mass Spectrometry

For analysis of EOs by gas chromatography–mass spectrometry, the samples before being injected into the GC system were dissolved in cyclohexane at the ratio of 1:100 (*v*/*v*). Further calculations were made considering the density of the specific oil recovered from plants *T. serpyllum* (LV–0.882 g/cm; C–0.934 g/cm), *T. vulgaris* (LV–0.919 g/cm; C–0.914 g/cm), and *S. montana* (LV–0.925 g/cm; C–0.941 g/cm). All preparation steps were performed at constant temperature in the laboratory glove portal box (25.0 °C).

### 4.2. Characterisation of Essential Oils Chemical Composition of by Gas Chromatography-Mass Spectrometry

The GC–MS analyses were done with a Clarus 580 system (PerkinElmer, Inc., Waltham, MA, USA), coupled to a Clarus SQ 8 C Mass Selective Detector (PerkinElmer, Inc., Waltham, MA, USA). An Omegawax 250 fused silica capillary (FSC) column (30 m × 0.25 mm, 0.25 μm film thickness) with a poly (ethylene glycol)-based stationary phase was used. The oven temperature was kept at 40 °C for 1.5 min, then programmed to 155 °C at a rate of 11 °C/min then raised at 185 °C for 8 min, and finally programmed to 230 °C at a rate of 15 °C/min and held at this point for a 1.3 min. The total separation time was 20 min. Helium (AGA, Jelgava, Latvia) was used as a carrier gas at the flow rate of 1.0 mL/min. Diluted samples (1/100 *v*/*v*, in cyclohexane) of 1μL were injected at 230 °C with a 1:10 split ratio (22 mL/min). Injector (glass wool-filled liner) and transfer line temperatures were set at 230 °C and 260 °C, respectively. The mass spectrometer operated at 70 eV with a mass range *m*/*z* 28 to 300 (multiplier 1700 V) and a scan time of 0.2 s (625 Da/sec). MS ion source temperature was 230 °C. The operation of the system is ensured by the TurboMass v6.0.0 user interface with NIST MS 2.2 Library (FairCom Corp., Columbia, MO, USA).

The concentration of volatile compounds present in the sample was determined by building a calibration curve of camphor (5.0–1000 µg mL^−1^). The content of thymol was determined separately by building a calibration curve of thymol. Linear least-squares regression of the peak areas as a function of the concentrations was performed to determine the correlation coefficients (R^2^ > 0.9990). The equation parameters (slope and intercept) of the standard curve were used to obtain the concentration values (camphor: y = 2.54 × 10^5^x − 6.35 × 10^5^; thymol: y = 2.11 × 10^5^x − 3.11 × 10^6^; y—peak area, µV·s; x —conc., µg·mL^−1^). The accuracy of the method was assessed by triplicate analysis of standard solutions at eight concentrations (5.0, 10.0, 50.0, 200.0, 400.0, 600.0, 800.0, and 1000.0).

The individual aroma volatile components were identified according to their retention index [62,63,64,65,66] and were compared with the reference spectra (Wiley and NIST databases). The retention index was determined experimentally from the retention time of n-alkanes under conditions of temperature-programmed chromatography using a two-functions calculation equation [67].

### 4.3. Antibacterial Assays

#### 4.3.1. *Escherichia coli* Strains

A total of 10 *E. coli* isolates were used to test the antibacterial properties of EOs. One of the isolates was used as antibiotic-sensitive reference strain ATCC 25922 (Bioscience, Botolph, United Kingdom), but the other isolates were collected from pig faeces according to the methodology described by [68]. Six isolates were ESBL-producing *E. coli*, containing all of three *bla* genes: *bla*_CTX-M_, *bla*_TEM_, and *bla*_SHV_. Three of ESBL-producing isolates were classified as MDR, which contained the following antimicrobial resistance profile: AM-CTX-CZ-AMC-GM-SXT-TMP-C-TE-ENO, and two with AM-CTX-CZ-AMC-GM-SXT-TMP-C-TE, but the other three as non-MDR ESBL-producing *E. coli.* Finally, the last three *E. coli* isolates did not show resistance to any of the 18 antibiotics [68].

#### 4.3.2. Determination of Minimum Inhibitory Concentration (MIC)

The antibacterial activity of EOs was determined by broth microdilution method as described earlier [69] with slight modifications. EOs were diluted with dimethyl sulfoxide (DMSO) (Sigma–Aldrich, Gillingham, UK) to a concentration of 500 μL/mL. The standardised bacterial suspensions were prepared using 20 h ± 2 h old *E. coli* cultures by suspending in peptone saline diluent (Maximum Recovery Diluent, Biolife, Milan, Italy) and adjusting optical density to 0.5 McF. The suspension was subsequently diluted to a concentration of approximately 1.5 × 10^6^ CFU/mL.

Subsequently, in 96-well plates, 100 μL of Muller–Hinton Broth (MHB) (Oxoid, Basingstoke, United Kingdom) was added to each well. Then, 100 μL of prepared solution of EO was added in the first well, followed by making a serial dilution. 50 μL of prepared bacterial inoculum and 50 μL saline (0.9% *w*/*v*) (in the test of EO with supplement, the saline solution part was replaced with hypertonic NaCl or CA solution, respectively) were added to all test wells. Each well eventually contained microbial suspension at a concentration of approximately 5 × 10^5^ CFU/mL. The total amount of aliquot was 200 μL per well and the final concentration of EO ranged from 125 to 0.000238 μL/mL.

MHB with bacterial inoculum plus saline was used as a positive control, while MHB alone was the negative control in each experiment. The inhibitory effect of DMSO was controlled on all *E. coli* strains. The concentration of DMSO at 6.25% was found to show no inhibitory activity and was below the toxic level as reported by [69].

To calculate minimum bactericidal concentration (MBC), before incubation of microplates, 1 μL of positive control aliquot was inoculated into two Muller–Hinton Agar II (MHA) (Biolife, Milan, Italy) plates for a baseline concentration of the bacteria used.

Finally, to prevent the evaporation of EOs during incubation and the probability of evaporating EOs falling into adjacent wells (carry-over effect), the top of the microplates before incubation was covered with parafilm (Biosigma, Venice, Italy) and lid. Microplates were incubated at 36 °C ± 1 °C for 24 h. After incubation, the MIC was defined as the lowest concentration capable of inhibiting bacterial growth (as evaluated by the absence of turbidity). Each experiment was repeated in duplicate.

#### 4.3.3. Determination of Minimum Bactericidal Concentration (MBC)

To determine the MBC, an aliquot of 1 μL of the represented MIC dilution, followed by four progressive concentration tests were subcultured into MHA for examining the bacterial growth. Plates were incubated at 36 °C ± 1 °C for 24 h and afterward, the number of colony-forming units (CFU) was determined. The MBC was calculated by the lowest concentration of EOs, resulting in killing 95% and 99.5% of the initial bacterial concentration.

#### 4.3.4. Determination of the Effects of EOs Supplementation with NaCl and Caprylic Acid on MICs and MBCs

To determine the effect of EOs supplementation either with 3% NaCl or 1 mM caprylic acid (CA) on MIC and MBC values, a 4-fold higher concentration stock solution was prepared for further assay.

An appropriate mass of NaCl (Sodium chloride, Sigma–Aldrich, Copenhagen, Denmark) was dissolved in deionised water to achieve a concentration of 12% *w*/*v* in the final solution, which was afterwards subjected to autoclaving. To reach 4 mM of CA (Sigma–Aldrich, Kuala Lumpur, Malaysia) solution, an appropriate amount of caprylic acid was weighed and dissolved in 20% ethanol–deionised water (*v*/*v*) solution.

The antibacterial activity of EOs supplemented with NaCl and CA was assessed following the protocol described above, though the part of 0.9% saline solution was substituted either with NaCl or CA. Each experiment was performed in duplicate. Simultaneously, the effect of 3% NaCl and 1 mM CA–ethanol solution on the bacteria growth inhibition and bactericidal effect of each *E. coli* strain were controlled.

### 4.4. Statistical Analysis

The components of the EOs were assessed by triplicate analysis, reported as mg/kg ± standard deviation (SD), and expressed as percentages (%). In vitro assays were performed in duplicate and values were expressed as mean ± standard error (SE). The effect of the origin EOs and plant species on antibacterial activity as well as the effect of the type of supplement and plant species of commercial EOs in the present study were analysed using two-way analysis of variance (ANOVA) by R Studio software (version 1.1.463). The Bonferroni post-hoc test was used to determine the level of significance. Paired sample *t*-test was conducted to determine the differences between value of MIC and MBC. An unpaired t-test was conducted to determine significant differences between the value of MIC against ESBL-producing and non-ESBL-producing *E. coli*, as well as against MDR *E. coli* and non-MDR *E. coli*. Differences were considered as significant if *p* < 0.05.

## 5. Conclusions

The concentration of total volatile aromatic compounds in the Lamiaceae family plants ranged from 2536.9–5041.9 mg/kg to 2388.7–5849.1 mg/kg in commercial and lab-scale produced EOs, respectively. The *p*-cymene, carvacrol, thymol, *γ*-terpinene, *β*-linalool, and *β*-caryophyllene were found as the main representatives of aroma volatiles detected in EOs, though concentrations varied depending on the EO group. The highest content of *p*-cymene was found in EOs recovered from *T. vulgaris*, with significant differences between the commercial SEU (35.40%) and lab-scale NEU (23.63%) samples. Carvacrol was another compound for which the relative abundancy was detected both in commercial (SEA) and lab-scale (NEU) *S. montana* and *T. serpyllum* EO samples, though, with considerable percentage fluctuations from 7.70% to 53.34%. Minor differences in the main aroma components were found between NEU and SEU EOs of *T. vulgaris* plant species. The content of thymol in NEU and SEU samples corresponded to 27.59% and 26.66%, respectively. The lowest content of thymol was found in EOs recovered from *T. serpyllum*, both for NEU (3.74%) and even lower in the commercial SEU sample (1.00%). The study revealed that perhaps the availability of non-oxygenated hydrocarbons at high concentrations in lab-scale EOs resulted in a marked reduction in antibacterial activity in comparison with those of commercial origin. It was also established that supplementation of commercial EOs with NaCl and CA greatly enhanced the antibacterial activity against *E. coli.* While reduced permeability and the presence of the efflux pump provided better resistance of ESBL-producing and MDR *E. coli* to most non-supplemented EOs compared to non-ESBL and non-MDR *E. coli*. However, the antibacterial activity of EOs towards ESBL and non-ESBL as well as MDR and non-MDR *E. coli* has been considerably increased by the enrichment of EOs with CA and NaCl especially. The highest antibacterial activity of EOs against high-level resistant *E. coli* was achieved by a combination of *T. vulgaris* with NaCl. Supplemented EOs with NaCl could be considered as a potential solution for suppressing the growth and viability of ESBL-producing and MDR *E. coli.* However, further studies are needed to clarify the risks of resistance development by the bacteria, especially to non-supplemented EOs.

## Figures and Tables

**Table 1:** Components of the essential oils (expressed in terms of camphor concentration).

RI	Compound Name	Composition, mg/kg ± SD, (%)					
		*Thymus vulgaris*		*Satureja montana*		*Thymus serpyllum*	
		C	LV	C	LV	C	LV
1015	*cis*-*p*-menthane ^1^	4.1 ± 0.1 (0.16)	-	-	-	-	-
1022	*α*-pinene ^1^	-	-	32.2 ± 0.5 (0.63)	50.7 ± 3.9 (0.99)	56.9 ± 3.0 (1.30)	20.8 ± 0.6 (0.35)
1023	***β*-terpinen ^1^**	**92.5 ± 4.6 (3.60)**	**21.4 ± 1.2 (0.87)**	-	-	-	-
1027	***α*-thujene ^1^**	**3.6 ± 0.2 (0.14)**	**35.8 ± 1.4 (1.46)**	**15.4 ± 0.5 (0.30)**	**99.6 ± 8.4 (1.94)**	**137.5 ± 12.6 (3.14)**	**40.9 ± 3.0 (0.70)**
1039	*trans*-*p*-menthane ^1^	4.2 ± 0.3 (0.17)	-	-	-	-	-
1058	*α*-fenchene ^1^	10.1 ± 0.5 (0.39)	-	-	-	-	-
1065	camphene ^1^	49.8 ± 2.0 (1.94)	15.9 ± 0.7 (0.65)	30.5 ± 0.6 (0.60)	20.0 ± 1.8 (0.39)	13.9 ± 0.7 (0.32)	17.9 ± 0.9 (0.30)
1107	*β*-pinene ^1^	12.6 ± 0.9 (0.49)	9.3 ± 0.6 (0.38)	9.4 ± 0.0 (0.19)	16.7 ± 1.2 (0.32)	17.1 ± 0.9 (0.39)	12.1 ± 0.6 (0.21)
1122	sabinene ^1^	-	5.8 ± 0.4 (0.24)	-	11.4 ± 0.4 (0.22)	4.8 ± 0.2 (0.11)	70.1 ± 3.0 (1.19)
1132	dehydrosabinene ^1^	-	-	t	t	t	-
1136	carvomenthene ^1^	6.8 ± 0.3 (0.26)	-	-	-	-	-
1149	*δ*-3-carene ^1^	-	4.9 ± 0.4 (0.24)	t	7.5 ± 0.5 (0.15)	9.9 ± 1.1 (0.23)	t
1165	***β*-myrcene + *α*-phellandrene ^1^**	**38.8 ± 3.4 (1.51)**	**54.3 ± 2.4 (0.20)**	**58.0 ± 1.3 (1.14)**	**172.8 ± 15.2 (3.36)**	**188.0 ± 17.7 (4.30)**	**201.7 ± 15.6 (3.43)**
1170	pseudolimonene ^1^	4.4 ± 0.4 (0.17)	-	-	-	-	-
1181	*α*-terpinene ^1^	11.2 ± 0.5 (0.44)	40.6 ± 2.2 (1.66)	70.3 ± 0.4 (1.39)	142.8 ± 11.0 (2.78)	96.8 ± 7.3 (2.21)	132.6 ± 6.6 (2.25)
1200	**limonene ^1^**	**91.1 ± 10.1 (3.55)**	**13.9 ± 0.6 (0.57)**	**16.4 ± 0.3 (0.32)**	**44.1 ± 3.0 (0.86)**	**18.8 ± 0.9 (0.43)**	**25.3 ± 0.7 (0.43)**
1210	1,8-cineole ^2^	42.8 ± 3.7 (1.67)	29.4 ± 1.9 (1.20)	30.6 ± 0.3 (0.60)	58.4 ± 6.3 (1.14)	40.5 ± 2.4 (0.93)	16.3 ± 0.7 (0.28)
1226	2-hexenal ^6^	-	-	-	t	-	-
1237	*β*-*cis*-ocimene ^1^	-	-	25.7 ± 0.7 (0.51)	17.0 ± 1.5 (0.33)	6.9 ± 0.6 (0.16)	58.9 ± 4.2 (1.00)
1249	***γ*-terpinene ^1^**	**149.0 ± 15.2 (5.81)**	**437.8 ± 16.3 (17.83)**	**336.7 ± 8.3 (6.64)**	**1152.1 ± 50.7(22.42)**	**404.4 ± 28.7 (9.24)**	**1291.0 ± 18.4 (21.93)**
1254	*β*-*trans*-ocimene ^1^	-	3.4 ± 0.1 (0.14)	5.9 ± 0.2 (0.12)	9.6 ± 3.2 (0.19)	6.8 ± 1.0 (0.16)	18.4 ± 5.7 (0.31)
1258	3-octanone ^6^	-	4.9 ± 0.1 (1.20)	-	-	-	11.3 ± 0.3 (0.19)
1275	***p*-cymene ^1^**	**908.5 ± 35.9 (35.4)**	**580.2 ± 22.6 (23.63)**	**660.1 ± 21.9 (13.02)**	**543.6 ± 42.6 (10.58)**	**358.0 ± 30.1 (8.18)**	**526.6 ± 29.7 (8.94)**
1286	terpinolene ^1^	-	6.0 ± 0.3 (0.25)	10.6 ± 0.1 (0.21)	10.7 ± 0.5 (0.21)	11.5 ± 0.8 (0.26)	25.0 ± 1.0 (0.42)
1308	*o*-cymene ^1^	4.3 ± 0.1 (0.17)	-	-	-	-	-
1320	(*Z*)-3-hexenyl acetate ^8^	-	3.0 ± 0.1 (0.12)	-	t	-	8.6 ± 0.3 (0.15)
1342	4-pentenyl butyrate ^8^	-	7.0 ± 0.3 (0.28)	-	-	-	-
1343	sulcatone ^6^	-	-	-	-	-	8.4 ± 0.1 (0.14)
1386	3-hexen-1-ol ^7^	-	7.6 ± 0.4 (0.31)	-	14.4 ± 1.3 (0.28)	t	-
1392	3-octanol ^7^	-	6.4 ± 0.5 (0.26)	6.0 ± 0.0 (0.12)	10.2 ± 0.5 (0.20)	11.2 ± 0.2 (0.26)	10.1 ± 0.5 (0.17)
1399	nonanal ^6^	-	-	-	-	-	t
1406	1-hepten-3-ol ^7^	-	-	-	t	-	-
1407	fenchone ^2^	3.4 ± 0.1 (0.13)	-	-	-	-	-
1432	photocitral B ^2^	-	-	-	-	-	t
1434	*β*-thujone ^2^	5.3 ± 0.3 (0.21)	-	-	-	-	-
1450	**1-octen-3-ol ^7^**	**-**	**28.9 ± 1.9 (1.18)**	**65.0 ± 1.2 (1.28)**	**161.5 ± 22.0 (3.14)**	**-**	**42.3 ± 1.5 (0.72)**
1449	*trans*-linalool furanoxide ^2^	4.0 ± 0.2 (0.16)	-	-		-	-
1453	*α*-thujone ^2^	3.7 ± 0.2 (0.14)	-	-		-	-
1468	*trans*-sabinene hydrate ^2^	3.3 ± 0.0 (0.13)	16.5 ± 1.0 (0.67)	26.4 ± 0.2 (0.52)	31.0 ± 3.7 (0.60)	-	60.7 ± 2.5 (1.03)
1475	*cis*-linalool furanoxide ^2^	3.9 ± 0.2 (0.15)	-	-	-	t	-
1477	isoledene ^4^	-	-	6.9 ± 0.7 (0.14)	-	-	-
1478	nerol oxide ^2^	-	-	-	-	-	t
1493	*α*-ylangene ^5^	-	-	t	-	-	-
1496	epiphotocitral A ^2^	-	-	-	-	-	t
1503	*α*-copaene ^5^	3.2 ± 0.1 (0.13)	-	10.9 ± 0.6 (0.21)	t	t	-
1507	decanal ^6^	-	-	-	-	-	t
1519	1-nonen-3-ol ^7^	-	-	t	5.6 ± 0.4 (0.11)	-	-
1531	*β*-bourbonene ^4^	-	-	10.1 ± 0.3 (0.20)	6.3 ± 0.9 (0.12)	-	19.2 ± 1.4 (0.33)
1533	camphor ^2^	8.2 ± 0.7 (0.32)	13.3 ± 0.7 (0.54)	5.9 ± 0.2 (0.12)	-	-	-
1542	*α*-gurjunene ^4^	-	-	t	-	-	-
1548	***β*-linalool ^2^**	**187.5 ± 13.9 (7.31)**	**88.0 ± 7.0 (3.58)**	**35.2 ± 1.0 (0.69)**	**103.5 ± 10.5 (2.01)**	**138.7 ± 10.0 (3.17)**	**11.2 ± 0.8 (0.19)**
1554	*cis*-sabinene hydrate ^2^	-	6.9 ± 0.5 (0.28)	13.6 ± 0.2 (0.27)	9.5 ± 0.7 (0.19)	14.8 ± 1.2 (0.34)	-
1555	isoneral ^2^	-	-	-	-	-	11.6 ± 1.6 (0.20)
1570	*trans*-*p*-menth-2-en-1-ol ^2^	-	4.4 ± 0.2 (0.18)	t	6.0 ± 0.2 (0.12)	5.3 ± 0.5 (0.12)	6.3 ± 0.3 (0.11)
1582	*p*-menth-3-en-1-ol ^2^	3.1 ± 0.1 (0.12)	-	-	-	-	-
1582	isogeranial ^2^	-	-	-	-	-	11.2 ± 0.5 (0.19)
1614	***β*-caryophyllene ^4^**	**42.3 ± 3.6 (1.65)**	**79.3 ± 4.9 (3.23)**	**728.6 ± 25.6 (14.37)**	**173.4 ± 17.4 (3.37)**	**149.7 ± 10.3 (3.42)**	**735.4 ± 26.9 (12.49)**
1586	*β*-ylangene ^4^		-	t	t	-	t
1593	bornyl acetate ^8^	4.6 ± 0.2 (0.18)	8.0 ± 0.3 (0.32)	9.6 ± 0.1 (0.19)	-	-	t
1602	methylthymol ^3^	-	28.9 ± 1.7 (1.18)	7.1 ± 0.1 (0.14)	t	-	69.1 ± 3.2 (1.17)
1606	*β*-copaene ^4^	-	-	t	t	t	t
1618	*β*-panasinsene ^4^	-	-	7.8 ± 2.3 (0.15)	-	-	-
1624	aromandendrene ^4^	-	-	48.5 ± 0.3 (0.96)	-	11.9 ± 1.0 (0.27)	-
1626	*trans*-dihydrocarvone ^2^	-	4.1 ± 0.1 (0.17)	-	-	8.6 ± 0.4 (0.20)	-
1632	selina-5,11-diene ^4^	-	-	t	-	-	-
1636	*cis*-p-menth-2-en-1-ol ^2^	-	-	-	t	-	t
1637	*p*-menth-8-en-1-ol ^2^	3.8 ± 0.1 (0.14)	-	-	-	-	-
1646	cis-dihydrocarvone ^2^	-	-	-	-	5.7 ± 0.1 (0.13)	-
1653	1-decen-3-ol ^7^	-	-	-	-	-	t
1663	allo-aromadendrene ^4^	-	-	9.5 ± 0.3 (0.19)	-	-	11.3 ± 0.7 (0.19)
1671	*trans*-*β*-farnesene ^4^	-	-	-	-	-	t
1674	cadina-1(6),4-diene ^4^	-	-	t	-	-	-
1678	isoborneol ^2^	3.6 ± 0.1 (0.14)	-	-	-	-	-
1680	lavandulol ^2^	-	4.4 ± 0.1 (0.18)	-	-	-	-
1681	*δ*-terpineol ^2^	-	-	-	t	-	-
1687	*α*-humulene ^4^	4.6 ± 0.2 (0.18)	4.4 ± 0.2 (0.18)	10.9 ± 0.2 (0.22)	6.7 ± 0.6 (0.13)	6.3 ± 0.4 (0.14)	20.2 ± 2.0 (0.34)
1696	*β*-citral ^2^	-	3.5 ± 0.1 (0.32)	-	-	-	111.6 ± 7.9 (1.90)
1703	*γ*-muurolene ^4^	-	-	18.3 ± 0.8 (0.36)	-	-	-
1703	*α*-terpineol ^2^	8.5 ± 0.5 (0.33)	7.9 ± 0.3 (0.32)	13.8 ± 0.4 (0.27)	14.1 ± 1.7 (0.27)	43.7 ± 2.7 (1.00)	11.1 ± 0.6 (0.19)
1708	*γ*-terpineol ^2^	3.2 ± 0.1 (0.13)	-	-	-	-	-
1712	**borneol ^2^**	**28.0 ± 2.7 (1.09)**	**28.9 ± 2.0 (1.18)**	**195.9 ± 2.9 (3.86)**	**56.7 ± 8.3 (1.10)**	**31.1 ± 1.9 (0.71)**	**12.3 ± 0.4 (0.21)**
1727	**germacrene D ^4^**	-	**6.1 ± 0.5 (0.17)**	-	**35.5 ± 2.9 (0.69)**	t	**178.8 ± 17.5 (3.04)**
1736	*β*-bisabolene ^4^	-	-	93.9 ± 2.7 (1.85)	38.5 ± 2.8 (0.75)	t	139.6 ± 15.0 (2.37)
1742	*α*-selinene ^4^	-	-	t	-	-	-
1746	*α*-citral ^2^	-	-	-	-	-	170.1 ± 16.7 (2.89)
1752	bicyclogermacrene ^4^	-	-	-	20.9 ± 1.8 (0.41)	15.6 ± 1.7 (0.36)	34.4 ± 6.6 (0.58)
1754	carvone ^2^	-	-	-	11.3 ± 0.8 (0.22)	10.2 ± 0.6 (0.23)	-
1755	nerol acetate ^8^	-	-	-	-	-	92.2 ± 7.9 (1.57)
1760	carvone ^2^	-	-	7.5 ± 0.1 (0.15)	-	-	-
1761	1-decanol ^7^	-	3.8 ± 0.2 (0.15)	-	-	-	-
1770	*δ*-cadinene ^4^	-	3.8 ± 0.1 (0.15)	25.5 ± 0.2 (0.50)	6.5 ± 0.2 (0.13)	6.0 ± 0.1 (0.14)	22.9 ± 1.8 (0.39)
1775	*γ*-cadinene ^4^	-	-	12.0 ± 0.6 (0.24)	t	t	t
1782	*trans*-α-bisabolene ^4^	-	-	t	t	-	-
1782	*β*-sesquiphellandrene ^4^	-	-	-	-	-	7.8 ± 0.3 (0.13)
1797	cubenene ^4^	-	-	t	-	-	-
1801	*p*-cumic aldehyde ^7^	-	-	t	-	-	-
1805	*cis*-geraniol ^2^	-	-	-	-	-	73.2 ± 6.8 (1.24)
1808	α-cadinene ^4^	-	-	t	-	-	-
1823	geranyl propionate ^8^	-	5.9 ± 0.1 (0.24)	-	-	-	-
1851	***trans*-geraniol ^2^**	-	**5.2 ± 0.2 (0.21)**	**6.2 ± 0.2 (0.12)**	-	-	**492.5 ± 15.9 (8.37)**
1856	*p*-cymen-8-ol ^7^	-	3.4 ± 0.1 (0.14)	7.9 ± 0.5 (0.16)	t	t	-
1861	thymyl acetate ^8^	-	3.7 ± 0.1 (0.15)	7.0 ± 0.4 (0.14)	-	-	-
1885	carvacryl acetate ^8^	-	-	10.1 ± 0.2 (0.20)	49.5 ± 4.6 (0.96)	t	t
1899	nerolidol ^5^	-	3.6 ± 0.2 (0.15)	-	-	-	6.6 ± 0.3 (0.11)
1890	epi-cubebol ^5^	-	-	t	t	-	-
1934	*α*-calacorene ^4^	-	-	t	-	-	-
1950	cubebol ^5^	-	-	-	-	-	t
2010	caryophyllene oxide ^5^	9.2 ± 0.6 (0.36)	12.8 ± 0.6 (0.52)	23.0 ± 0.1 (0.45)	11.2 ± 0.5 (0.22)	7.9 ± 0.5 (0.18)	51.7 ± 5.3 (0.88)
2044	*trans*-nerolidol ^5^	-	-	-	-	-	109.6 ± 7.8 (1.86)
2065	germacrene D-4-ol ^5^	3.2 ± 0.3 (0.12)	3.6 ± 0.1 (0.15)	-	-	-	30.8 ± 2.6 (0.52)
2082	epi-cubenol ^5^	-	-	-	-	-	t
2093	globulol ^5^	-	-	t	-	t	-
2101	viridiflorol ^5^	-	-	t	-	-	-
2106	*p*-cymen-7-ol ^3^	-	-	t	-	-	-
2118	spathulenol ^5^	-	-	17.0 ± 0.5 (0.34)	9.1 ± 0.6 (0.18)	9.3 ± 0.1 (0.21)	9.9 ± 0.3 (0.17)
2143	**thymol ^3^**	**684.2 ± 36.9 (26.66)**	**677.6 ± 36.3 (27.59)**	**867.3 ± 18.1 (17.10)**	**72.6 ± 5.9 (1.41)**	**43.9 ± 2.7 (1.00)**	**220.3 ± 13.7 (3.74)**
2149	tau-muurolol ^5^	-	-	-	-	-	10.8 ± 0.8 (0.18)
2163	*δ*-cadinol ^5^	-	-	-	-	-	t
2183	**carvacrol ^3^**	**78.9 ± 6.2 (3.07)**	**34.7 ± 2.2 (1.41)**	**1337.3 ± 8.4 (26.37)**	**1826.0 ± 69.1 (35.54)**	**2334.4 ± 46.1 (53.34)**	**453.1 ± 22.3 (7.70)**
2219	*α*-cadinol ^5^	-	-	-	-	-	21.6 ± 1.4 (0.37)
2311	caryophylladienol I ^5^	-	-	-	-	-	t
2316	caryophylladienol II ^5^	-	-	-	-	-	t
**Total identified**		2536.9 (99.70)	2388.7 (99.14)	5041.9 (99.91)	5090.2 (99.59)	4270 (99.6)	5849.1 (99.77)
monoterpene hydrocarbons ^1^		1391.0 ± 74.4	1229.6 ± 49.3	1280.5 ± 35.1	2302.3 ± 144	1335.2 ± 105.9	2446.4 ± 90.2
oxygenated monoterpenes ^2^		312.4 ± 22.9	216.7 ± 14.2	339.7 ± 6.2	308.8 ± 28.5	302.5 ± 20.2	1008.4 ± 56.5
aromatic monoterpenoids ^3^		766.4 ± 43.1	749.7 ± 40.2	2233.8 ± 27	1906.1 ± 75	2382.8 ± 48.8	742.6 ± 39.3
sesquiterpene hydrocarbons ^4^		50.1 ± 4.0	93.7 ± 5.7	1029.8 ± 37.1	308.8 ± 28.5	209.6 ± 14.8	1188.5 ± 72.9
oxygenated sesquiterpenes ^5^		12.4 ± 0.9	20.1 ± 0.9	51.3 ± 0.9	23.7 ± 1.2	21.4 ± 0.8	263.8 ± 19.7
aliphatic aldehydes & ketones ^6^		-	4.9 ± 0.1	4.4 ± 0.1	4.5 ± 0.1	-	30.7 ± 0.7
aliphatic alcohols ^7^		-	46.7 ± 3.1	75.9 ± 1.4	191.8 ± 24.1	14.7 ± 0.4	57.3 ± 2.1
esters ^8^		4.6 ± 0.2	27.5 ± 0.9	26.6 ± 0.8	53.6 ± 4.8	3.8 ± 0.9	111.5 ± 8.6
Thymol *		**836.1 ± 44.4**	**828.0 ± 44.0**	**1056.1 ± 21.8**	**100.0 ± 7.1**	**65.36 ± 3.3**	**278.41 ± 16.5**

Note: * Calculated by external calibration of thymol. RI: retention index calculated (temperature-programmed chromatography) against C9–C26 *n*-alkanes on the Omegawax 250 capillary column. The concentration of volatiles was expressed using the obtained calibration curve for the external standard of camphor. %: Data in brackets was the percentage of the tested volatile in the total volatile concentration. C: commercial oils, LV: hydrodistillation oils of native plants (Latvian). Text in bold indicates dominant individual compounds detected in EOs samples. Superscript 1–8: Molecular class: monoterpene hydrocarbons (1); oxygenated monoterpenes (2); aromatic monoterpenoids (3); sesquiterpene hydrocarbons (4); oxygenated sesquiterpenes (5); aliphatic aldehydes and ketones (6); aliphatic alcohols (7); esters (8), t–traces (≤0.1%).

**Table 2:** Minimum inhibitory concentration (MIC) and minimum bactericidal concentration (MBC at 95% and 99.5%) of Latvian and commercial essential oils against reference *E. coli* (ATCC 25922) strain and faecal *E. coli* isolated from pigs.

Essential Oils		MIC μL/mL		MBC 95% μL/mL		MBC 99.5% μL/mL	
		Faecal *E. coli*	ATCC 25922	Faecal *E. coli*	ATCC 25922	Faecal *E. coli*	ATCC 25922
**Commercial (SEU)**	*T. serpyllum*	0.209 ± 0.0464	0.488 ± 0.0000	0.209 ± 0.0464	0.488 ± 0.0000	0.209 ± 0.0464	0.488 ± 0.0000
	*T. vulgaris*	0.148 ± 0.0200	0.079 ± 0.0202	0.148 ± 0.0200	0.079 ± 0.0202	0.148 ± 0.0200	0.079 ± 0.0202
	*S. montana*	0.073 ± 0.0137	0.006 ± 0.0031	0.076 ± 0.0144	0.006 ± 0.0031	0.076 ± 0.0144	0.006 ± 0.0031
**Latvian (NEU)**	*T. serpyllum*	1.004 ± 0.1149	1.465 ± 0.2819	1.221 ± 0.1134	1.465 ± 0.2819	1.221 ± 0.1134	1.465 ± 0.2819
	*T. vulgaris*	0.590 ± 0.0713	0.977 ± 0.0000	0.644 ± 0.0763	0.977 ± 0.0000	0.658 ± 0.0733	0.977 ± 0.0000
	*S. montana*	0.278 ± 0.0434	0.366 ± 0.1221	0.278 ± 0.0434	0.367 ± 0.1221	0.278 ± 0.0434	0.366 ± 0.1221

## Data Availability

The data presented in this study are available on request from the corresponding author.
